# Protein hydrolysate and oil from fish waste reveal potential as dog food ingredients

**DOI:** 10.3389/fvets.2024.1372023

**Published:** 2024-04-22

**Authors:** Ana R. J. Cabrita, Margarida R. G. Maia, Ana P. Alves, Tiago Aires, Ana Rosa, André Almeida, Rui Martins, António J. M. Fonseca

**Affiliations:** ^1^REQUIMTE, LAQV, ICBAS, School of Medicine and Biomedical Sciences, University of Porto, Porto, Portugal; ^2^SORGAL, Sociedade de Óleos e Rações, S.A., Lugar da Pardala, S. João Ovar, Portugal; ^3^SEBOL, Comércio e Indústria de Sebo, S.A., Santo Antão do Tojal, Portugal; ^4^Indústria Transformadora de Subprodutos, S.A., Herdade da Palmeira—Olheiros do Meio—São José da Lamarosa Agolada Coruche, Coruche, Portugal

**Keywords:** fish hydrolysate, fish oil, functionality, nutrient digestion, palatability, pet food

## Abstract

The increased fish consumption by the growing human population in the world translates into an increase in fish waste. The reintroduction of these fish by-products into food and feed chains presents economic benefits and contributes to counteracting their negative environmental impact. Under this context, the present study aimed to evaluate the effects of the dietary inclusion of fish hydrolysate and oil obtained from fish waste (experimental diet) in substitution of shrimp hydrolysate and salmon oil (control diet) mainly imported from third countries on palatability, apparent total tract digestibility, fecal characteristics and metabolites, blood fatty acid profile, flatulence, and coat quality of adult dogs. A two-bowl test was performed to evaluate palatability by the pairwise comparison between the two diets. A feeding trial was conducted according to a crossover design with two diets (control and experimental diets), six adult Beagle dogs per diet, and two periods of 6 weeks each. The replacement of shrimp hydrolysate and salmon oil with fish hydrolysate and oil did not affect the first diet approach and taste, as well as the intake ratio. Generally, the digestibility of dry matter, nutrients, and energy was not affected by diet, but the intake of digestible crude protein (CP) and ether extract was higher, respectively, with the control and the experimental diet. The higher intake of eicosapentaenoic acid and docosahexaenoic acid with the experimental diet was reflected in a higher content of these long-chain polyunsaturated fatty acids and the omega-3 index of red blood cells, but it did not affect coat quality. The significantly higher intake of digestible CP with the control diet might have contributed to the higher fecal ammonia-N and valerate concentrations. Daily fecal output and characteristics were similar between diets. Overall, results suggest that fish hydrolysate and oil from the agrifood industry might constitute sustainable functional ingredients for dog feeding while adding value for wild fisheries, aquaculture, and fish farming under a circular economy approach and reducing dependence on imports from third countries with a high carbon footprint.

## 1 Introduction

In the last decades, the increase in the world human population, which is increasingly consuming more fish and fish products due to their role as part of a healthy diet, has been the main driver for the growth in total fisheries and aquaculture (1). Indeed, recent estimates set an expected world fish production of 201 million tons by 2030, comprising an overall increase of 12.8% from 2018 to 2020, with an increasing share from aquaculture (2). The observed increase in fish consumption is translated into an increase in fish waste, being the majority of lost or unused biomass (e.g., skin, bones, heads, viscera, and trimmings) found during filleting and preparation of products for the final consumer (3). However, these by-products have the potential to originate high-value products that can be reintroduced into food and feed chains due to their richness in nutrients and bioactive compounds, such as proteins, bioactive peptides, polyunsaturated fatty acids (PUFAs), minerals, and enzymes (4, 5). Taking advantage of fish by-products has not only economic benefits but also counteracts the negative environmental impact of improper fish waste disposal, namely through the release of organic compounds into aquatic environments (5, 6). Indeed, the valorization of fish by-products is commonly considered the preferred option to manage fish waste, whereas when important pollutants are present, waste management processes should determine the fate of fish by-products (7). The application of the circular economy concept to the fishery field increases the rate of reuse and decreases the underutilization of fish waste, thus contributing to a reduction of the pressure on natural resources (3, 8), which is aligned with the Circular Economy Action Plan (9) launched by the European Union as part of the European Green Deal. Protein hydrolysates and oils rich in omega-3 PUFA, such as eicosapentaenoic (C20:5 n-3, EPA) and docosahexaenoic (C22:6 n-3, DHA) acids, are among the most common functional feed ingredients obtained from fish by- and co-products (BCPs) with great interest, among others, in companion animal feeding due to the current trends observed in this sector (6). Indeed, along with a growing worldwide pet population (10), owners are increasingly favoring sustainable functional ingredients that not only ensure energy and nutrient requirements but also benefit the health and wellbeing of their animals, contributing to a decrease in the risk of chronic disease development and an increase in life expectancy (11).

In this context, *in vitro* and *in vivo* studies, namely with dogs, have shown that protein hydrolysates provide amino acids more easily absorbed in the small intestine (12), with numerous biological activities such as hypoallergenic (13), anti-stress (14, 15), satiety (16), antihypertensive, antithrombotic, immunomodulatory, and antioxidative effects (17), as well as acting as palatability enhancers (13, 18). However, despite the high interest in protein hydrolysates and their use in commercial diets, studies on companion animals are scarce, especially with fish hydrolysates.

Regarding the long-chain omega-3 PUFA, EPA and DHA are daily recommended for fully-grown (19), growing, and reproductive dogs (19–21), being also considered to play a role in disease prevention and used in several therapeutic diets (e.g., cardiovascular, renal, gastrointestinal, orthopedic, and dermatological) at higher doses (22–26). Fish oil is considered the primary source of EPA and DHA (27), but due to sustainability reasons and also taking into consideration the Sustainable Development Goal of the United Nations Agenda 2030 to prevent overfishing, alternative sources of fish oil obtained from farmed and wild fish are required. Although several marine sources [e.g., microalgae, macroalgae, krill, copepods, single-cell organisms; (28, 29)] and genetically modified plant oilseeds [e.g., rapeseed, thale cress, and camelina; (30–33)] have been evaluated as EPA and DHA sources, the extraction of oil from fish waste originated from the processing stage might constitute a sustainable approach that also has the advantage of being able to be applied locally, contributing to a lower environmental impact. Therefore, the present study aimed to evaluate the effects of the inclusion of locally produced fish hydrolysate and oil obtained from fish waste in the substitution of imported shrimp hydrolysate (mainly from Ecuador, the major producer of shrimp) and salmon oil (mainly from the North of Europe, Norway, and Scotland, the main producers of salmon), on palatability, apparent total tract digestibility, fecal characteristics and metabolites, blood fatty acid profile, and coat quality of adult dogs.

## 2 Materials and methods

Trials were approved by the Animal Ethics Committee of the School of Medicine and Biomedical Sciences, University of Porto, and licensed by the Portuguese General Directorate of Food and Veterinary Medicine (Permit No 0421/000/000/2021). Trained scientists in laboratory animal science (FELASA, category C) conducted the experiments in line with good animal welfare practices (European Union Directive 2010/63/EU).

### 2.1 Animals and housing

Twelve healthy adult Beagle dogs (2 intact males, 4 neutered males, and 6 spayed females), 5.4 ± 0.57 years old, weighing 11.8 ± 2.20 kg with a body condition score of 4.3 ± 0.69 (assessed according to a 9-point scale; (34)), were used in the experimental trials. Dogs were clinically examined before the start of the trials to ensure their suitability to participate in the studies. At the kennel of the School of Medicine and Biomedical Sciences, University of Porto, animals were housed in pairs in environmentally enriched and communicating boxes comprising an interior and an exterior area of 1.8 and 3.5 m^2^, respectively, with sliding doors for their individual feeding. Animals were allowed to exercise and socialize in an outdoor park area under supervision and were leash-walked once a day for at least 30 min. To ensure the collection of individual feces, animals were housed individually during the collection period, and supervised access to the outdoor park was maintained between daily meals.

### 2.2 Diets and feeding

Fish hydrolysate and oil were supplied by a company group (Empresa Transformadora de Subprodutos Animais S.A., ETSA, Loures, Portugal) dedicated to recycling in the food sector and were obtained from fish by-products comprising heads, tails, skin, slices, and whole fish mainly of salmon, sea bream, sea bass, and redfish. These category 3 animal by-products are, according to Regulation European Commission 1069/2009 (35), identified as the lowest risk group since they come from healthy slaughtered animals and, despite not being fit for human consumption, present no risk to animals or the environment. Enzymatic hydrolysis was carried out on minced fish by-products with the non-specific serine endopeptidase from *Bacillus licheniformis* Alcalase 2.4 L (Novozymes^®^, Bagsvaerd, Denmark) in a ratio enzyme/substrate of 0.2%, during 4 h at 65 ± 5°C at pH ± 8, ensured by adjustment with 1 N NaOH. Once completion of the hydrolysis, heat treatment was applied at approximately 90°C for 10 to 15 min to deactivate the enzyme, and the liquid was filtered through a 450 μm sieve (200 mm diameter, VWR International, Oregon, United States) to separate residues such as scales and bones that had not been hydrolyzed from the aqueous fraction. The hydrolyzed liquid was centrifuged for 15 min at 5,394 × g at room temperature in a Gyrozen 1,248 centrifuge (Gyrozen^®^ Co., Ltd., South Korea) to remove impurities, resulting in three layers: (i) the lipid fraction, from which fish oil was obtained; (ii) the mineral fraction; and (iii) the protein fraction that was further concentrated (four times) in a KNF RC900 Rotary Evaporator (KNF^®^, Breisgau, Germany). After this concentration phase, the protein hydrolysate was centrifuged again for 5 min at 5,394 × g at room temperature to ensure that it was free of possible suspended solids (such as the presence of some minerals and residues) and then subjected to the spray drying process (Mini Spray Dryer B-290, Büchi, Switzerland) at 130°C, 95% aspiration, and 13% system flow rate.

A commercial diet for adult medium-size dogs (Sorgal Pet Food, Ovar, Portugal) with the inclusion of 5% shrimp hydrolysate (Symrise Aqua, Equator) and 3% salmon oil (Symrise Aqua, Norway) was used as the control diet. An experimental diet was formulated by Sorgal (Ovar, Portugal) according to the current formulation matrixes to be iso-energetic, iso-lipidic, and iso-nitrogenous, using the same ingredients of the control diet, with slight adjustments on wheat grain (6.0 and 7.5% for the control and experimental diet), pea concentrate (7.0 and 5.0%), and poultry fat (5.0 and 5.3%), and the replacement of 5% shrimp hydrolysate with 5% fish hydrolysate, and 3% of salmon oil with 3.2% of fish oil.

Animals were individually fed the daily ration in two equal meals, at 8:30 a.m. and 17:00 p.m., with free access to fresh water. Both control and experimental diets were fed as an extruded kibble. Daily food allowance was calculated according to the body condition score (34) and the ideal body weight to meet the metabolizable energy (ME) requirements (19).

### 2.3 Palatability trial

A two-bowl test (36) was performed to evaluate palatability by pairwise comparison between the two diets. Animals (*n* = 12) were given the choice between the two diets in two different bowls (45 cm apart) on both meals for two consecutive days. The sample size and the duration of the trial were defined according to an earlier study (37) that found significant differences in intake ratio among diets without or with the inclusion of shrimp hydrolysate. The position of the bowls was switched between meals to eliminate any bowl-placement bias. The amount of food offered in each bowl was calculated as half of the daily ration needed to ensure the ME requirements of dogs (19). Diet first approached and diet first tasted were recorded. The test ended after 30 min or until animals consumed all the food available in one bowl. To calculate the intake ratio of both diets, food offered and food residues were weighed.

### 2.4 Feeding trial design and sample collection

Dogs were divided into two groups, blocked for sex and whether the males were intact or neutered, and received the control and experimental diets in two consecutive experimental periods following a crossover arrangement (six dogs per diet and period, following the recommendation of FEDIAF (19) for digestibility trials). Each experimental period lasted for 6 weeks. Daily food intake was recorded throughout the trial. Dogs were weighed, and their body condition was assessed every 2 weeks to adjust the daily food allowance. Measurements and sample collection were performed in the last week of each period. Dogs were subjected to a physical examination, including urinalysis, performed on urine samples collected directly into sterilized containers by voluntary urination before the morning meal. On one day of the last week of each period, blood was collected from each dog via jugular into clot activator gel tubes (EDTA BD Vacutainer; VWR International, Carnaxide, Portugal) for the analysis of the fatty acid profile of red blood cells (RBCs). Dogs were fasted overnight prior to blood collection but had free access to water at all times. In 5 days of the last week of each experimental week, total feces excreted by dogs were collected to determine the apparent total tract digestibility of dry matter (DM), nutrients, energy, and dietary ME content. During these days, the number of defecations was recorded every day, and individual fresh samples, collected within 15 min after defecation, were scored using a 5-point scale from 1 (watery diarrhea) to 5 (powdery hard mass pellets) (38). Samples were weighed, mixed, subsampled at different locations, and immediately frozen at −20°C in individual containers for later analysis of the chemical composition, fecal pH, ammonia-N, and volatile fatty acid concentrations carried out in mixed feces composited by period and dog.

### 2.5 Analytical procedures

#### 2.5.1 Diets and feces

Diets and fecal samples were dried in a forced-air oven at 65°C until constant weight. Ground (1 mm) diet and feces samples were analyzed in duplicate according to official methods (39), determining the contents of DM (ID 934.01), ash (ID 942.05), Kjeldahl nitrogen (N) (in dried diet samples and fresh feces samples; ID 990.03), and ether extract (EE, ID 920.39). Crude protein (CP) was calculated as Kjeldahl N × 6.25. For diet and feces samples, neutral detergent fiber (with α-amylase and without sodium sulfite, NDF) was determined and expressed exclusive of residual ash (40), starch content was analyzed in 0.5 mm samples according to Salomonsson and Theander (41), and gross energy (GE) was determined using an adiabatic bomb calorimeter (Werke C2000, IKA, Staufen, Germany).

Fatty acids of the control and experimental diets were converted to fatty acid methyl esters by acid-catalyzed transesterification with methanolic HCl (42) and analyzed by gas chromatography, as reported by Maia, Fonseca (43). Nonadecanoic acid (Matreya LLC, Pleasant Gap, PA) was used as an internal standard, and the identification of fatty acids was performed by comparing retention times to commercially available standards (Supelco 37 Component FAME Mix, PUFA No.1, PUFA No.2, PUFA No.3, Sigma-Aldrich Co. LLC, St. Louis, MO, United States of America).

For amino acid analysis, determined as described by Aragão, Cabano (44), samples of the control and experimental diets were hydrolyzed with 6 M HCL solution at 116°C for 48 h and precolumn derivatized with Waters AccQ Fluor Reagent (6-aminoquinolyl-N-hydroxysuccinimidyl carbamate) flowing the AccQ Tag method (Waters, Milford, MA). Analyses were performed in ultra-high-performance liquid chromatography on a Waters reversed-phase amino acid analysis system. Norvaline was used as an internal standard, and the resulting peaks were analyzed with EMPOWER software (Waters).

#### 2.5.2 Urine

Color and turbidity were evaluated as recommended by Alleman and Wamsley (45). Urinalysis was performed with a dipstick, and pH and density, respectively, determined with a potentiometer (pH and Ion-Meter GLP 22, Crison, Barcelona, Spain) and a refractometer (URIVET hand refractometer, HPM003, Zuzi).

#### 2.5.3 Blood

RBCs were separated from plasma by centrifugation at 3,000 rpm for 15 min at 4°C and fatty acids transesterified as previously described by Harris, Pottala (46). The resulting fatty acid methyl esters were analyzed by gas chromatography using a Shimadzu GC-2010 Plus (Shimadzu Corporation, Kyoto, Japan) equipped with a capillary column (SP-2560, 100 m × 0.25 mm × 0.20 μm; Supelco, Bellefonte, PA, United States of America) and a flame-ionization detector. The oven’s initial temperature of 50°C was held for 1 min, then increased at a rate of 50°C/min to 150°C and held for 20 min, then increased at 1°C/min to 190°C and held for 3 min, and further increased at 1°C/min to 220°C and held for 20 min. Injector and detector temperatures were set at 250 and 280°C, respectively. Hydrogen was the carrier gas at a linear velocity of 17.2 cm/s. Fatty acids were identified by comparing retention times to standards (Supelco 37 Component FAME Mix, BAME Mix, PUFA No.1, PUFA No.2, PUFA No.3, Sigma-Aldrich, St. Louis, MO, United States of America; GLC-110 Mixture, Matreya, Pleasant Gap, PA, United States of America). The omega-3 index was calculated as the sum of EPA and DHA expressed as a proportion of total identified fatty acids.

### 2.6 Fecal pH and metabolites

Feces were thawed, and pH was determined using a potentiometer (pH and Ion-Meter GLP 22, Crison, Barcelona, Spain). Ammonia-N concentration was determined according to the method of Smith and Murphy (47) adapted to dog feces as described by Cabrita, Guilherme-Fernandes (48). Briefly, 1 g of feces were solubilized in 10 mL of KCl 2 M, centrifuged for 60 min at 5200 × g at 4°C, and the supernatant filtered using a 0.45 μm pore size polyethersulfone syringe filter (FILTER-LAB, Barcelona, Spain). Forty μL of supernatant were then mixed with 40 μL of water, 2.5 mL of phenol solution, and 2 mL of alkaline hypochlorite solution. After incubation for 10 min at 37°C and 40 min in the dark at 22°C, the absorbance of samples was read at 550 nm in a SynergyTM HT Multimode plate reader (BioTek^®^ Instruments Inc., Winooski, VT). An ammonia solution (32 mg/dL) was used as a standard. Volatile fatty acid analysis was performed by gas chromatography in the supernatant from feces acidified with an orthophosphoric acid solution and centrifuged for 15 min at 2,360 × g at 4°C as described by Pereira, Maia (49).

### 2.7 Evaluation of flatulence

The assessment of dogs’ flatulence was performed for 5 h (from 10:30 h until 15:30 h) as described by Pereira, Guedes (50), using a device resembling the one described by Collins and Perez-Camargo (51). Briefly, a dog vest was developed to hold the device hardware, which consists of an air pump fitted with an H_2_S sensor connected to a microcontroller powered by a power bank. Gases were collected from the anal area through a 55 cm-long plastic tube (inner diameter 0.5 cm) connected to the pump-sensor box and an O-ring placed at the base of the tail. The H_2_S concentrations were presented in parts per million and recorded every 2 s. The number of flatulence episodes, maximum, and average intensity of H_2_S detected in each episode were recorded. Due to the sensitivity of the sensor and the possibility of cross-reactivity with other gases, blank data were collected in the room where the measures took place for 5 h to distinguish flatulence episodes from noise. Hydrogen sulfide values higher than 5 ppm and lasting at least for 10 s were considered the threshold for flatulence detection.

### 2.8 Evaluation of coat quality

Taking as reference the number of elements (7) used in the study of Hester (52), and in order to represent both the average dog owners and individuals involved in pet health, a naïve panel of eight dog owners (two kennel staff, one animal science post-doctoral researcher, one laboratory technician, two animal science students, and two veterinarians), blind to the treatments, performed a sensory assessment of the coat condition. A day before the evaluation, all dogs were washed using a hypoallergenic dog shampoo. The panel assessed glossiness, greasiness, softness, and scale as defined by Marsh, Ruedisueli (53), using the 5-point scale proposed by Hester (52), in which 5 is the best and 1 is the worst score (Table 1).

**Table 1:** Five-point scale used for coat scoring, adapted from Hester (52).

	Glossiness	Greasiness	Softness	Scale	Overall coat quality
1	Very dull (poorest, no shine at all)	Very greasy (poorest)	Very brittle (poorest)	Very scaly (poorest)	Poor (very dull, brittle, dry, scaly, or greasy) (poorest)
2	Moderately dull	Moderately greasy	Moderately brittle	Moderately scaly	Fair
3	Slightly shiny	Mildly greasy	Slightly soft	Mildly scaly	Good
4	Moderately shiny	Minimally greasy	Moderately soft	Minimally scaly	Very Good
5	Very shiny (best, a lot of shine)	Not greasy (best)	Very soft (best)	No scale (best)	Excellent (very shiny, very soft, no scale, not greasy) (best)

### 2.9 Calculations and statistical analysis

First-approach and first taste results from the palatability trial were submitted to the chi-square test and the intake ratio to the Student’s *t*-test, both at a 5% probability level.

Fecal production (%) was calculated as:


$$\mathrm{Fecal production}\;\left(\%\right)=\frac{\mathrm{dried feces output}\;\left(\frac{g}{d}\right)}{\mathrm{dry}\;\mathrm{matter intake}\;\left(\frac{g}{d}\right)}\times 100$$

Diet apparent total tract digestibility (%) was calculated as follows:


$$\mathrm{ATTD}\;\left(\%\right)=\frac{\mathrm{nutrient intake}\;\left(\frac{g}{d}\right)-\mathrm{fecal output}\;\left(\frac{g}{d}\right)}{\mathrm{nutrient intake}\;\left(\frac{g}{d}\right)}\times 100$$

The diet ME content was calculated according to Hall, Melendez (54) using the following equation:


$$\mathrm{ME}\;\left(\frac{MJ}{\mathrm{kg}\;DM}\right)=\frac{\begin{array}{l} \left(GE\;\mathrm{intake}\;\left(\frac{MJ}{d}\right)-\mathrm{fecal}\;GE\;\left(\frac{MJ}{d}\right)\right)-\\ \left(CP\;\mathrm{intake}\;\left(\frac{g}{d}\right)-\mathrm{fecal}\;CP\left(\frac{g}{d}\right)\right)\times 5.23 \end{array}}{DM\;\mathrm{intake}\;\left(\frac{g}{d}\right)}$$

Data from the feeding trial were analyzed using the mixed procedure of the Statistical Analysis Systems software package (SAS 2021, release 3.1.0., SAS Institute, Cary, NC, United States of America). The model included diet, period, and dietary sequence as fixed effects and animal within dietary sequence as a random effect. When differences were significant (*p* < 0.05), the Tukey test was used to compare means. The level of significance was set at a *p*-value of <0.05, and a trend was considered for a *p*-value of <0.10.

## 3 Results

### 3.1 Chemical composition of diets

The proximate composition of the studied diets is presented in Table 2. The control diet presented higher CP and slightly lower EE contents than the experimental diet, with NDF, starch, and GE contents similar between diets. The experimental diet presented a higher percentage of n-3 PUFAs, particularly EPA, docosapentaenoic acid (DPA, C22:5 n-3), and DHA, and a lower percentage of n-6 PUFA than the control diet, thus presenting a lower n-6:n-3 ratio (Table 3). Total amino acid content and profile were similar between diets (Table 4).

**Table 2:** Proximate composition (g 100 g^−^^1^ dry matter, DM) and gross energy (MJ kg^−^^1^ DM) of the control and experimental diets.

	Diet^a^	
	Control	Experimental
DM, %	95.7	97.1
Ash	8.77	7.60
Crude protein	29.1	27.0
Ether extract	10.4	11.6
Neutral detergent fiber	11.2	11.8
Starch	31.4	32.2
Gross energy	18.1	18.8

a Extruded complete diet with the inclusion of shrimp hydrolysate and salmon oil (control) or with fish hydrolysate and fish oil (experimental).

**Table 3:** Fatty acid content (g 100 g^−^^1^ dry matter) and profile (g 100 g^−^^1^ total fatty acids) of the control and experimental diets.

	Diet^a^	
	Control	Experimental
Total fatty acids	8.26	10.1
C12:0	ND^b^	0.079
C14:0, %	0.804	1.49
C14:1 *cis*-9, %	0.094	0.090
C15:0, %	0.166	0.182
C16:0, %	22.1	21.2
C16:1 *cis*-9, %	3.76	3.98
C17:0, %	0.203	0.200
C18:0, %	6.28	5.91
C18:1 *trans*-9, %	ND	0.090
C18:1 *cis*-9, %	33.0	32.8
C18:1 *cis*-11, %	1.78	2.06
C16:4 *n*-1, %	ND	0.099
C18:2 *n*-6, %	25.8	20.8
C20:0, %	0.224	0.247
C18:3 *n*-6, %	0.131	0.131
C20:1 *cis*-9, %	ND	0.149
C20:1 *cis*-11, %	0.409	1.08
C18:3 *n*-3, %	2.32	2.15
C20:1 *cis*-13, %	ND	0.053
C18:4 *n*-3, %	0.197	0.482
C20:2 *n*-6, %	0.261	0.284
C22:0, %	0.182	0.173
C20:3 *n*-6, %	0.150	0.150
C22:1 *cis*-11, %	0.185	1.04
C22:1 *cis*-13, %	ND	0.151
C20:4 *n*-6, %	0.574	0.568
C20:4 *n*-3, %	ND	0.175
C20:5 *n*-3, %	0.355	1.25
C24:0, %	0.175	0.185
C24:1 *cis*-15, %	0.084	0.172
C22:4 *n*-6, %	0.117	0.092
C21:5 *n*-3, %	ND	0.067
C22:5 *n*-6, %	ND	0.083
C22:5 *n*-3, %	0.116	0.360
C22:6 *n*-3, %	0.494	1.96
OBCFA^c^	0.369	0.382
SFA^d^	30.2	29.6
MUFA	39.4	41.7
PUFA	30.5	28.7
PUFA *n*-3	3.48	6.44
PUFA *n*-6	27.0	22.1
*n*-6:*n*-3	7.76	3.44

a Extruded complete diet with the inclusion of shrimp hydrolysate and salmon oil (control) or with fish hydrolysate and fish oil (experimental).

b ND, not detected.

c OBCFA, odd and branched-chain fatty acids.

d SFA, saturated fatty acids.

e MUFA, monounsaturated fatty acids.

f PUFA, polyunsaturated fatty acids.

**Table 4:** Amino acid content (g 100 g^−^^1^ dry matter) and profile (g 100 g^−^^1^ total amino acids) of the control and experimental diets.

	Diet^a^	
	Control	Experimental
Total amino acids	26.9	26.2
Essential amino acids		
Arginine	7.03	6.30
Histidine	2.50	2.40
Lysine	6.71	6.61
Threonine	4.39	4.26
Isoleucine	5.12	4.98
Leucine	7.77	7.56
Valine	5.45	5.21
Methionine	2.27	2.30
Methionine + cystine	5.28	5.27
Phenylalanine	5.33	5.00
Phenylalanine + tyrosine	9.37	8.70
Non-essential amino acids		
Cystine	3.02	2.96
Tyrosine	4.04	3.70
Aspartic acid + Asparagine	9.64	9.08
Glutamic acid + Glutamine	15.37	15.10
Alanine	6.00	6.11
Glycine	7.08	7.28
Proline	6.13	6.25
Serine	5.06	4.89

a Extruded complete diet with the inclusion of shrimp hydrolysate and salmon oil (control) or with fish hydrolysate and fish oil (experimental).

### 3.2 Palatability trial

The replacement of shrimp hydrolysate and salmon oil with fish hydrolysate and oil did not affect the first diet approach (*p* = 0.773) or taste (*p* = 0.386), as well as the intake ratio (*p* = 0.971; Table 5).

**Table 5:** First approach and taste, and intake ratio of the control and experimental diet.

	Diet^a^		*p*-value
	Control	Experimental	
First approach	25	23	0.773
First taste	27	21	0.386
Intake ratio	49.9	50.1	0.971

a Extruded complete diet with the inclusion of shrimp hydrolysate and salmon oil (control) or with fish hydrolysate and fish oil (experimental).

### 3.3 Feeding trial

#### 3.3.1 Body weight, food intake, and fecal output

None of the dogs refused to eat any of the diets, and all remained healthy through the trial, with no signs of illness or maldigestion, with urine pH, density, and urinalysis results within the reference values for healthy animals (45) and not differing between diets (data not shown). Table 6 presents the body weight, body condition score, food and nutrient intake, and fecal output of dogs fed the control and experimental diets. Body weight and body condition score were not affected by diet (*p* > 0.05). Similarly, diet did not affect DM, nutrient, and energy intake (*p* > 0.05) with the exceptions of higher EE, EPA, DHA, and arachidonic acid (ARA, C20:4 n-6) intake with the experimental diet (*p* < 0.05) and a higher CP (*p* = 0.006) intake with the control diet. Daily fecal output was similar between diets (*p* > 0.05).

**Table 6:** Body weight, body condition score, food and nutrient intake, and fecal output of dogs fed the control and experimental diets.

	Diet^a^		SEM	*p*-value
	Control	Experimental		
Body weight, kg	12.1	12.0	0.67	0.680
Body condition score	4.3	4.3	0.17	0.395
Food intake				
g d^−1^ (as-is)	186.2	182.5	11.79	0.406
g d^−1^ (dry matter, DM, basis)	178.3	177.0	11.36	0.772
MJ d^−1^	3.22	3.33	0.210	0.220
Nutrient intake, g d^−1^				
Organic matter	162.6	163.6	10.44	0.814
Crude protein	51.9	47.8	3.18	0.006
Ether extract	18.5	20.5	1.26	0.003
Neutral detergent fiber	19.7	20.9	1.28	0.079
Starch	56.0	57.0	3.61	0.465
C22:6 n-3	0.068	0.331	0.0158	<0.001
C20:5 n-3	0.050	0.215	0.0103	<0.001
C22:6 n-3 + C20:5 n-3	0.119	0.547	0.026	<0.001
Fecal output				
g d^−1^ (as is)	135.2	149.0	13.09	0.175
g d^−1^ (DM basis)	37.1	40.3	3.15	0.244
MJ d^−1^	0.513	0.566	0.0443	0.197
Fecal production, %	21.1	22.9	1.37	0.321
Defecations, no. d^−1^	1.57	1.68	0.090	0.211

a Extruded complete diet with the inclusion of shrimp hydrolysate and salmon oil (control) or with fish hydrolysate and fish oil (experimental).

#### 3.3.2 Apparent total tract macronutrient digestibility and intake of digestible nutrients

Apparent total tract digestibility of DM, nutrients, and energy was not affected by diet (*p* > 0.05), except for NDF (*p* = 0.009), which was higher in the control diet (Table 7). Conversely, dietary ME content was higher in the experimental diet (*p* = 0.034). The intake of digestible DM and macronutrients was similar between diets, with the only exception of the intake of digestible CP and EE, which were higher in the control and the experimental diet (*p* < 0.001).

**Table 7:** Apparent total tract macronutrient digestibility (ATTD), estimated dietary metabolizable energy content and digestible dry matter, and nutrient intake of dogs fed the control and experimental diets.

	Diet^a^		SEM	*P*-value
	Control	Experimental		
ATTD, %				
Dry matter (DM)	79.6	77.1	1.43	0.122
Organic matter	83.6	81.7	1.10	0.212
Crude protein	75.4	72.8	1.68	0.284
Ether extract	95.5	95.2	0.35	0.469
Starch	99.6	99.4	0.07	0.070
Neutral detergent fiber	63.5	53.5	2.20	0.009
Energy	83.8	82.8	1.09	0.478
ME^b^, MJ kg^−1^ DM	14.0	14.5	0.18	0.034
Intake^c^, g d^−1^				
DM	142.2	136.8	9.59	0.315
Organic matter	136.3	134.0	9.17	0.632
Crude protein	39.2	35.0	2.68	0.030
Ether extract	17.7	19.6	1.21	0.005
Starch	55.8	56.7	3.61	0.513
Neutral detergent fiber	12.6	11.3	0.92	0.153

a Extruded complete diet with the inclusion of shrimp hydrolysate and salmon oil (control) or with fish hydrolysate and fish oil (experimental).

b ME, metabolizable energy.

c Digestible DM and macronutrient intake.

#### 3.3.3 Fecal characteristics and metabolites

Fecal characteristics, including pH, scores, DM, and metabolite concentrations, are presented in Table 8. None of the measured parameters were affected by diet (*p* > 0.05) except for ammonia-N (*p* = 0.042) and valerate concentration, which were higher (*p* = 0.007) in feces from dogs fed the control diet (Table 8).

**Table 8:** Fecal characteristics and metabolites of dogs in the control and experimental diets.

	Diet^a^		SEM	*p*-value
	Control	Experimental		
Fecal characteristics				
pH	6.01	5.98	0.066	0.674
Fecal score^b^	3.14	3.27	0.125	0.129
Dry matter, DM, feces (%)	28.1	28.1	0.73	0.941
Fecal metabolites				
Ammonia-N, mg/kg DM	283.2	239.4	13.99	0.042
Volatile fatty acids, μmol/kg DM				
Total	0.479	0.467	0.0185	0.493
Acetate	0.291	0.281	0.0091	0.364
Propionate	0.111	0.119	0.0083	0.323
*Iso*-butyrate	0.006	0.005	0.0003	0.131
Butyrate	0.046	0.042	0.0031	0.187
*Iso*-valerate	0.008	0.007	0.0005	0.095
Valerate	0.016	0.012	0.0014	0.007
*Iso*-caproate	0.001	0.001	0.0001	0.540
Caproate	0.001	0.001	0.0001	0.543

a Extruded complete diet with the inclusion of shrimp hydrolysate and salmon oil (control) or with fish hydrolysate and fish oil (experimental).

b Fecal scores: 1, watery, liquid that can be poured; 2, soft, unformed, stool assumes the shape of the container; 3, soft, formed, moist, softer stool that retains shape; 4, hard, formed, dry stool, remains firm and soft; and 5, hard, dry pellets, small, hard mass.

#### 3.3.4 Flatulence

The flatulence assessment is presented in Table 9. The number of episodes detected in 5 h averaged four, regardless of the diet. The average and maximum intensity of episodes (H_2_S ppm detected) ranged from 8.98 to 12.07 ppm and from 14.0 to 28.6 ppm, respectively, with no significant differences being detected between diets (*p* > 0.05).

**Table 9:** Number of episodes, average, and maximum intensity (H_2_S detected) of flatus of dogs fed the control and experimental diets.

	Diet^a^		SEM	*p*-value
	Control	Experimental		
Number of episodes	3.75	3.92	0.569	0.812
Average intensity, ppm	12.07	8.98	3.227	0.515
Maximum intensity, ppm	28.6	14.0	9.11	0.283

a Extruded complete diet with the inclusion of shrimp hydrolysate and salmon oil (control) or with fish hydrolysate and fish oil (experimental).

#### 3.3.5 Fatty acid profile of red blood cells

The experimental diet promoted the contents of EPA (*p* = 0.037) and DHA (*p* = 0.007), and, consequently, the omega-3 index (*p* = 0.005; Table 10) of the RBCs.

**Table 10:** Fatty acids’ profile (g 100 g^-1^) and omega-3 index of red blood cells from dogs fed the control and experimental diets.

	Diet^a^		SEM	*p*-value
	Control	Experimental		
C16:0	16.4	16.1	0.64	0.441
C18:0	37.4	38.4	1.13	0.259
C18:1 *cis*-9	9.37	9.29	0.365	0.847
C18:1 *cis*-11	2.43	2.42	0.203	0.987
C18:2 *n*-6	10.7	10.3	0.370	0.183
C20:4 *n*-6	21.0	19.7	1.55	0.145
C20:5 *n*-3	1.41	2.02	0.181	0.037
C22:6 *n*-3	1.17	1.75	0.165	0.007
Omega-3 index^b^	2.58	3.77	0.234	0.005

a Extruded complete diet with the inclusion of shrimp hydrolysate and salmon oil (control) or with fish hydrolysate and fish oil (experimental).

b Calculated as C20:5 *n*-3 + C22:6 *n*-3.

#### 3.3.6 Coat quality

The effect of diet on visual and sensorial coat evaluation is presented in Table 11. Scores were 3.99 vs. 3.83, 4.04 vs. 4.08, 4.04 vs. 3.98, 4.06 vs. 3.88, and 4.08 vs. 3.99 for glossiness, greasiness, softness, scale, and overall coat quality for the control and experimental diets. None of the parameters were affected by diet (*p* > 0.05).

**Table 11:** Visual and sensorial coat evaluation of dogs fed the control and experimental diets.

	Diet^a^		SEM	*p*-value
	Control	Experimental		
Glossiness	3.96	3.83	0.126	0.301
Greasiness	4.04	4.08	0.065	0.613
Softness	4.04	3.98	0.120	0.594
Scale	4.06	3.88	0.144	0.399
Overall coat quality	4.08	3.99	0.083	0.436

a Extruded complete diet with the inclusion of shrimp hydrolysate and salmon oil (control) or with fish hydrolysate and fish oil (experimental).

## 4 Discussion

The use of fish BCPs from the agrifood sector for the extraction of oil rich in omega-3 fatty acids and protein hydrolysates constitutes a sustainable solution to the increasing problem of fish waste disposal. If applied locally, this circular economy further contributes to lowering dependency on third countries and mitigating the environmental impact of the food sector. For the full exploitation of these potential high-value products, *in vivo* studies are needed. Therefore, this current study aimed to investigate the effects of fish hydrolysate and oil from fish wastes during the processing stage on palatability, apparent total tract digestibility, fecal characteristics and metabolites, blood fatty acid profile, and coat quality of adult dogs. The experimental diet containing fish hydrolysate and oil was compared to a commercial diet including shrimp hydrolysate, commonly used in fish diets (55, 56), but recently shown to have the potential to be included in dog diets (37), and salmon oil, a common oil source used in dog food. With shrimp hydrolysate and salmon oil mainly imported from third countries, the use of fish hydrolysate and oil locally produced might contribute to the increased sustainability of the pet food sector.

Both diets used in the current study ensured the energy and nutrient requirements of adult dogs, according to FEDIAF (19). The chemical composition of the diets reflected the composition of the hydrolysates and oils used, with the main differences being observed in ash, CP, and EE contents, and fatty acid profiles. The lower ash content of the experimental diet might be a favorable aspect of the selection of the studied sources for addition in high-digestibility diets, as a high ash content may compromise protein digestibility (57). Regarding the EE content and fatty acid profile, it is well known that these parameters can be affected by fish species, size, age, sex, fish parts, diet, extraction method, and commercial source. The contents of omega-3 long-chain PUFA, namely EPA, DPA, and DHA, are generally higher in oil from marine than freshwater fish species (58–60). *In vitro* and *in vivo* studies with dogs showed that these conditionally essential fatty acids (19) play important metabolic roles in cell membrane fluidity, neural development, cognitive status, visual acuity, and inflammatory and immunologic responses (61–63). Similarly, the CP content and amino acid profile of seafood and crustacean hydrolysates might differ depending on the primary source of protein (species and part of the animal), enzyme source, and hydrolysis conditions (17, 64). An earlier study (37) showed that in shrimp hydrolysate obtained from the enzymatic hydrolysis of heads and cephalothoraxes of *Litopenaeus vannamei*, as the one used in the current study, lysine and leucine were the essential amino acids found in the highest amounts, and glutamic acid plus glutamine were the main non-essential amino acids. For fish hydrolysates, the review from Chalamaiah, Kumar (65) reported aspartic acid and glutamic acid to be found in high amounts in most of the fish protein hydrolysates, and that muscle, head, skin, and visceral hydrolysates contain all the essential and non-essential amino acids, whereas in fish frame protein hydrolysates, aromatic amino acids were not reported.

In the present study, diet palatability was evaluated using the two-bowl test, considered effective when evaluating intentional product enhancements but recognized to be unable to detect eventual flavor fatigue, longer-term nutritional feedback on taste and satiety, and dependent on the reference diet (36). Additionally, the number of animals included in the test is an important consideration when interpreting the results. Although using 20 animals over 2 or 4 days is considered to increase the test resolution (36), the number of animals used in palatability tests greatly differs in the literature, with studies reporting the use of 8 (66), 10 (67), and 16 (68) animals. In the current study, despite the sample size (12 animals) being defined according to an earlier study that evaluated the dietary supplementation with shrimp hydrolysate (37), the number of animals used was lower than the generally recommended (20 animals), so some caution should be taken when interpreting the results obtained. Animals accepted well both the control and the experimental diet, with no differences in the first approach, taste, or intake ratio. It is well known that factors such as food flavor, texture, ingredients, chemical composition, temperature, previous experience of the animal, and size and shape of the kibble can affect palatability (69), and this constitutes an important aspect when formulating diets as palatability might affect both food intake and the provision of nutrients and strongly determine food repurchase potential. In the current study, both diets presented the same kibble size, shape, and texture. Although animal protein hydrolysates are among the most common palatability enhancers used in commercial pet food, scientific studies are scarce (70). Sensorial characteristics of hydrolyzed proteins are associated with the raw protein source and mixture of peptides (71), with short peptides and amino acids such as taurine, glycine, arginine, glutamic acid, and alanine acting as feeding stimulants for companion animals (13). Moreover, bitterness was earlier associated with the molecular weight of peptides, with reports of increased bitterness in soy protein hydrolysates when the molecular weight of the peptides ranged from 4 to 2 kDa and decreased bitterness with peptides <1 kDa (72). Despite these reports, several studies showed the high palatability of protein hydrolysates, such as fish (18) and chicken (73) hydrolysates for dogs.

Dietary fat content is also known to affect palatability, with dogs preferring high-fat diets (74, 75). Being unsaturated fatty acids, particularly EPA and DHA, highly susceptible to deterioration (76), the free radical formation and breakdown of hydroperoxides release organic substances such as short-chain aldehydes, ketones, and alcohols, seen as off-flavors, which might promote the rejection of the food (77, 78). Although not measured in the current study, the stability of the oils used might not have constituted an issue, as no differences were observed on the palatability test, and both diets were well accepted during the feeding trial.

The daily food allowance was determined to ensure energy requirements and adjusted according to the ideal body weight and body condition score, and thus these parameters remained constant during the study. As diets presented similar energy content and digestibility, the daily amount of food required to ensure the requirements did not vary between diets. The differences found in the intake of CP, EE, and some fatty acids reflect the different chemical compositions of the diets. These differences were not reflected in the fecal output and stool quality that were adequate for dogs fed both diets and constitute important parameters for pet owners. An earlier study with dogs found higher fecal volume and lower fecal DM content with the dietary inclusion of 258 g/kg of hydrolyzed chicken liver when compared to a control diet with poultry byproduct, bovine meat, and bone meal, that might be explained by the high osmolarity of that attracts water to the intestinal lumen, but fecal DM output and fecal scores were not affected (79).

Digestibility of DM, nutrients, and energy was similar between diets except for NDF digestibility, which was higher in the control diet but did not affect the intake of digestible NDF, fecal pH, total VFA production, acetate concentration, or ammonia-N concentration. The differences in the dietary EE content and fatty acid profile might contribute to explaining the observed effects on NDF digestibility among diets, namely through the modulation of fecal microbial populations (80, 81). Although no significant effects were observed on CP digestibility, the significantly higher intake of digestible CP observed with the control diet might have contributed to the higher fecal ammonia-N and valerate concentrations (and a tendency for a higher iso-valerate concentration), suggesting that a higher amount of undigested protein has reached the intestine with this diet. Indeed, ammonia-N is generated by the deamination of amino acids, being toxic at high concentrations, and branched-chain fatty acids originate from the fermentation of branched-chain amino acids (82, 83). Although not herein measured, the results obtained might have affected fecal odor that results from the formation of several putrefactive substances from the colonic fermentation of endogenous and undigested amino acids, such as ammonia, aliphatic amines, branched-chain fatty acids, indoles, phenols, and volatile sulfur-containing compounds, that may also exert adverse effects on colonic health (84, 85). While large individual variation inhibited the significance of differences in H_2_S emissions, the higher values obtained with the control diet might suggest a higher availability of sulfur-amino acids in the colon of dogs fed the control diet. Using diets not differing on CP digestibility (70.1 to 73.7%), but differing on Zn source (Zn proteinate and Zn sulfate), and without or with the addition of a solid-state fermentation product of *Aspergillus niger* with residual enzymatic activity, Pereira, Guedes (50) also found a high individual variation on the number of episodes (3.3–10.3), average (11.0–17.0 ppm), and maximum intensity (20.4–33.8 ppm) of adult Beagle dogs. Further studies are needed to confirm the effect observed as H_2_S inhibits the utilization of butyrate by colonocytes caused by mitochondrial metalloproteins and is associated with ulcerative colitis (86) as well as the severity of flatus malodor (51). Additionally, despite the scarcity of information available, the study performed by Zhao and McCamish (87) with intact and hydrolyzed soy protein intestinally perfused in increasing amounts in dogs with duodenal and midintestinal fistulas has shown that intestinal transit is slowed in a load-dependent fashion regardless of the source of protein, but it is more pronounced with intact protein, and that a greater amount is absorbed in the proximal half of the small intestine when protein is delivered in a hydrolyzed form, also concluding that as digestion is the rate-limiting step of assimilation in the small intestine, a longer residence time might be needed with a higher protein load. Despite these observations, no effects were observed on fecal output or characteristics.

As the digestibility of EE did not differ between diets, the higher intake of digestible EE reflected the higher EE intake. Differences in EPA and DHA intake were further reflected in the EPA and DHA of RBC and the omega-3 index. Indeed, the RBC omega-3 index has been reported as a biomarker of long-term EPA and DHA intake in humans (88, 89), being inversely correlated with neutrophil–lymphocyte ratio (a systemic inflammation biomarker, 90), cardiovascular disease risk, and acute death (90). Recently, Harris, Jackson (91) suggested its use as a health status biomarker in dogs. Although no range has yet been established for optimal health status in dogs, a 0.3 to 7.0% omega-3 index was determined in routine veterinary dog blood samples, with higher values being associated with improved health (91). An increased omega-3 index was also reported in the RBC of dogs fed a diet supplemented with krill meal or fish meal plus fish oil for 4 weeks (92) and krill oil for 6 weeks (93). The highest values were reported for krill oil (2.70%) and krill meal (2.36%), in the range of those reported for the control diet (2.58%). Moreover, the experimental diet promoted an increase in the omega-3 index by approximately 1.5-fold compared with the control diet, suggesting a potential health-promoting effect of fish BCP’s inclusion in pet food, namely on promoting cardiovascular function and anti-inflammatory activity.

The increased absorption and metabolization of EPA and DHA by dogs fed the experimental diet were hypothesized to promote the coat quality of dogs, as suggested by Combarros and Castilla-Castano (94), but no differences were observed by a näive panel in the present study. The lack of effects may be due to the similarity of the oils used, both rich in omega-3 PUFA, and the use of healthy dogs. Indeed, Logas and Kunkle (95) reported an improvement in the coat quality of dogs with skin diseases and poor coat quality after 6 weeks of supplementation with EPA-rich marine oil (180 mg EPA/120 mg DHA) compared with corn oil supplement; no differences were observed after 3 weeks. More recently, Combarros, Castilla-Castano (94) investigated the effect of a fish oil supplement (110 mg EPA/68 mg DHA) for 3 months on dogs with poor hair coats and observed a reduced clinical score after 2 months of EPA and DHA supplementation when compared to placebo-supplemented dogs, but no differences after 4 weeks. These results support the need for longer studies to fully unveil the potential of fish by-product hydrolysate and oil supplementation on the coat quality of healthy dogs.

## 5 Conclusion

The present study shows that a diet with the inclusion of locally produced fish oil and hydrolysate from the agrifood industry in place of imported salmon oil and shrimp hydrolysate was well accepted by dogs, not affecting food intake, digestibility, or fecal characteristics. The lower fecal ammonia-N and valerate concentrations suggest a lower amount of undigested protein reaching the small intestine or a higher protein absorption. Conversely, fish hydrolysate and oil promoted blood EPA, DHA, and omega-3 index, suggesting a potential health-promoting effect. Overall, the results support the potential of fish hydrolysate and oil from the agrifood industry to be used in dog food, contributing to increasing the sustainability of the pet food sector. Further research is needed to evaluate the functional role of these fish by-products.

## Data availability statement

The raw data supporting the conclusions of this article will be made available by the authors, without undue reservation.

## Ethics statement

The animal study was approved by Animal Ethics Committee of School of Medicine and Biomedical Sciences, University of Porto, and licensed by the Portuguese General Directorate of Food and Veterinary Medicine (Permit No 0421/000/000/2021). The study was conducted in accordance with the local legislation and institutional requirements.

## Author contributions

AC: Supervision, Writing – original draft, Resources, Project administration, Investigation, Funding acquisition, Formal analysis, Conceptualization. MM: Writing – review & editing, Investigation, Formal analysis, Conceptualization. APA: Writing – review & editing, Investigation. TA: Writing – review & editing, Resources. AR: Writing – review & editing, Resources. AA: Writing – review & editing, Resources. RM: Writing – review & editing, Resources. AF: Writing – review & editing, Resources, Investigation, Funding acquisition, Formal analysis, Conceptualization.
